# Supplementary material for: Invasive Californian death caps develop mushrooms unisexually and bisexually
Source: Nat Commun. 2023 Oct 24;14:6560. doi: 10.1038/s41467-023-42317-z (PMC10598064; doi:10.1038/s41467-023-42317-z)
Supplement: Supplementary file 1 — Supplementary Information [file 41467_2023_42317_MOESM1_ESM.pdf]

Supplementary Information

Table of Contents

Supplementary Notes *2*
Supplementary Figures.. *8*
Supplementary Tables... *24*
Supplementary Discussion.. *29*
Supplementary References *31*

Supplementary Notes

1. Heterozygosity estimation, k-mer analysis and allele frequency

The estimated heterozygosities of most individuals are normally distributed with a range between 1.7×10^{-3} and 3.7×10^{-3}, but two outliers have estimated heterozygosities of a different order of magnitude, at 3.07×10^{-4} (g21) and 2.5×10^{-4} (g22). Both of these individuals are from Drake2, PRNS, California. Two sporocarps of individual g21 were found in 2014. One sporocarp of individual g22 was found in 2004 and five were found in 2014. (We collected both g21 and g22 again in 2021, but none of the genomes of the 2021 specimens were sequenced.)

Most heterozygotic individuals with a sequencing depth higher than 50x exhibit a typical pattern of k-mer and allele frequency distributions: in a k-mer frequency distribution, heterozygous individuals have a minor peak at half of the depth of major peak. In an allele frequency distribution, heterozygous individuals have a peak at 0.5. The most deeply sequenced sporocarps of the two individuals with low estimated heterozygosities have sequencing depths of 71.1x (g21) and 67.6x (g22), but neither have typical k-mer or allele frequency distributions, additional evidence they are homokaryotic. While four other individuals (g9, g10, g11 and g12) with sequencing depths higher than 50x also do not have visible minor peak in k-mer frequency distributions, the absence of minor peak in these individuals is likely caused by greater sequencing error: the frequencies of k-mers at 0.5 normalized k-mer depth of these four individuals were higher than frequencies of g21 and g22 (Fig. 1b).

In allele sequencing frequency plots, all heterozygotic individuals, including the four individuals without visible minor peaks in k-mer graphs, show a peak at 0.5. By contrast, the two individuals with low estimated heterozygosities display no peak, again suggesting they are homokaryotic (Fig. 1c). The allele sequencing frequency plots using the filtered VCF without the

re-calling of variants generated nearly identical results to the plots generated using the filtered VCF with re-calling.

2. Kinship analysis

Two genetic individuals (four mushrooms collected in 2004 and 2015) are the parent or offsprings of g21 and six individuals (made up of 22 mushrooms collected in 1993, 2004, 2014, 2015) are the parent or offsprings of g22 (Supplementary Fig. 1b,c). Because only one individual can be the parent of a homokaryotic individual, both g21 and g22 must be mating. We assume the population is not intensively inbreeding: significant inbreeding would cause a significant reduction in heterozygosity, which was absent in the individuals identified as parent or offsprings (Fig. 1 and Supplementary Data 1). We note that while heterokaryotic individual g24 and the homozygous g22 share a high kinship (0.462), because most other heterokaryotic parent/offsprings of homokaryotic individuals had much higher estimated kinships, we took a conservative approach and do not consider g24 as a parent/offspring of g22.

3. Protein structural analysis

PR proteins are membrane proteins, which usually have signal peptides, and belong to the GPCR family which is characterized by seven transmembrane helices. Both of *A. phalloides*' PR proteins encoded seven transmembrane helices, but neither of them had signal peptides. Instead of signal peptides, they may encode signal anchor sequences for translocating the proteins to membrane[1]. Structural models from AlphaFold2 also support a folding of the transmembrane domains into a tertiary structure resembling the structure of a typical GPCR (Supplementary Fig. 6).

In the HD1 from each allele, we identified a NLS at 310–360 amino acid, but no NLS was predicted in any HD2. Homeodomains were found at 100–175 amino acid in HD1 and 140–200 amino acid in HD2. The tertiary homeodomain structures predicted by AlphaFold-Multimer exhibited a high structural similarity with the mating type HD of *Saccharomyces cerevisiae*. AlphaFold-Multimer also predicted low predict alignment errors (PAEs) among amino acids in the N-terminal of the two proteins suggesting a high confidence of them being binding sites for heterodimerization (Supplementary Fig. 11). However, the two homeodomains do not fit the quaternary structure of the mating type HD of *Saccharomyces cerevisiae* (PDB Acc. No. 1LEB [https://doi.org/10.2210/pdb1LE8/pdb]), probably because of the low confidence/flexible linkers between the N-terminal domains and homeodomains.

4. Interspecies phylogeny of *PR* genes

The species-tree-aware gene phylogeny suggests the two PRs in *A. phalloides* are orthologous to non-mating type PRs in other agaricomycetes (Supplementary Fig. 8). However, the orthologs in non-agaricomycete species may be mating type determining PRs. For example, the ortholog of *Ap.00g075660* in *Cryptococcus neoformans* is a mating type determining PR (*Cpr2*, or, *CND05800*; Supplementary Fig. 9). The different functions of these genes in different classes of fungi may be the result of a duplication of the two pheromone receptors at the ancestral branch of agaricomycetes, followed by neofunctionalization in the new gene copies.

5. Pheromone identification

Two hypothetical genes encoding putative pheromones were identified on two contigs (4 and 135) and both had conserved -CaaX motifs (Supplementary Fig. 7). However, only one hypothetical pheromone precursor had the ER/DR motif commonly found in pheromone

precursors of model species[2]. The phenomenon is also observed in close relatives of the death cap, including *A. muscaria* var. *guessowii* and *V. volvacea*, which also do not have any ER/DR motifs in any putative pheromone precursors[3,4].

6. *De novo* genome assemblies used to identify *HD* genes at intraspecies level

In the genome assemblies of most sporocarps there were two HD unitigs, but two and eleven sporocarps had three and one HD unitigs, respectively (Supplementary Data 1). The genomes of the two sporocarps with three HD unitigs have a low sequencing coverage (34.5x and 9.5x). Both specimens belong to heterokaryotic, multi-sporocarp genetic individuals, and other sporocarps of the same individuals possess two HD unitigs. The eleven samples with one HD unitig included all eight homokaryotic sporocarps, and three other putatively heterokaryotic sporocarps (10019, 10169 and 10233), each with a very low coverage (12.0–26.2x). HD unitigs of 10019 and 10169 were very short: 1,892 and 495 bp, whereas the HD unitig of 10233 was longer: 24,659 bp. Further mapping of raw reads back to the HD assemblies of 10019 and 10233 show heterozygosity at the locus, but we could not map raw reads back to the 10169 assembly. But we did not detect any heterozygosity at any HD locus in any homokaryotic sporocarp. We consider the results for 10019, 10169 and 10233 as caused by shallow sequencing.

Remaining sporocarps present two HD unitigs: 35, 28 and five of their genome assemblies form closed, open and complexed bubbles, respectively, and the two HD unitigs of the other five samples were detached (Supplementary Data 1). Specimens belonging to the same genetic individual can present differently, for example, three genomes with detached HD unitigs were assembled into closed, open or complexed bubbles in other sporocarps of the same genetic individuals. Two HD unitigs representing different alleles of the HD locus appeared to be present in most if not all individuals.

7. Annotation of *HD* genes

Annotations of the *HD* genes of sporocarps collected between 2004 and 2015 from California and Portugal identify 22 unique *HD1* alleles and 21 unique *HD2* alleles, which translate to 20 and 18 unique protein products, respectively. We excluded specimens 10233 and 10288 from analyses because of problems with the genome assemblies for the two specimens. The lengths of HD1 and HD2 proteins range from 532 to 563, and from 385 to 406, amino acids. The genes contain two and three introns, respectively, with intron lengths of 48 to 62, and 45 to 69, bp.

8. Yeast two hybrid of *HD* genes

The ORFs of the *HD1* and *HD2* alleles 5, 8, and 13 were fused to the *GAL4* activation domain (AD) or *GAL4* DNA binding domain (BD) creating either "prey" or "bait" constructs, which were transformed into *S. cerevisiae* in all possible combinations. Interactions were determined by assessing multiple reporter genes, both qualitatively using filter assays and selective media (Supplementary Fig. 12a), and quantitatively using liquid β-galactosidase assays (Supplementary Fig. 12b). We found that HD1 from each allele when fused to the Gal4 BD resulted in autoactivation and expression of the reporters (Supplementary Fig. 12a, lines 8, 10, 12, and S11B, lines 11, 15, 19). Furthermore, addition of an HD2 fused to the Gal4 AD did not increase reporter expression levels in these strains (Supplementary Fig. 12b, lines 11-22), suggesting that potential interactions between the bait and prey proteins could be obscured by the BD-HD1 background activation. In contrast, HD1s fused to the Gal4 AD did not autoactivate the reporters (Supplementary Fig. 12a, lines 2, 4, 6) and were therefore used to assess interactions with HD2s fused to the Gal4 BD.

To test the possibility that the HD1 or HD2 from allele 13 could auto-interact and form homodimers to regulate homokaryotic sexual development, we tested all combinations of BD-HD1 + AD-HD1 alleles and BD-HD2 + AD-HD2 alleles (Supplementary Fig. 13). As before, all BD-HD1 alleles were auto-activating and did not appear to change in the presence of any AD-HD1 alleles (Supplementary Fig. 13, lines 1-9), precluding an assessment of possible HD1-HD1 interactions. In contrast, we found that no combinations of BD-HD2 + AD-HD2 alleles induced expression of the reporter genes, indicating that HD2 of alleles 5, 8, and 13 are unlikely to form regulatory homodimers.

Supplementary Figures

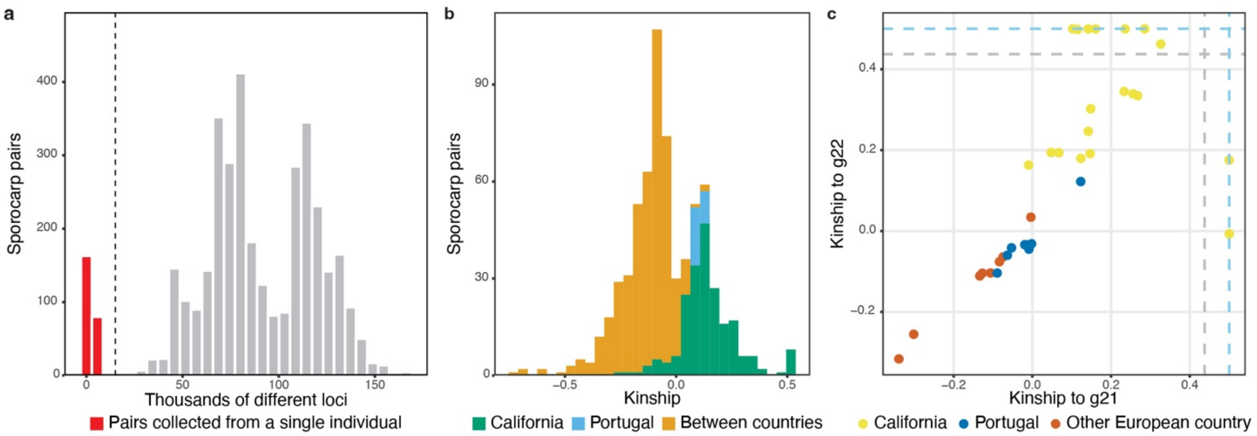

Supplementary Fig. 1. Clone-correction and evidence for mating between homokaryotic individuals (g21 and g22) and heterokaryotic individuals. (a) Numbers of SNPs differentiating pairs of sporocarps. Note distinct peak at left (in red). Each of the sporocarp pairs in the peak represents two mushrooms collected from a single genetic individual. (b) Kinship estimates between pairs of individuals from California, pairs of individuals from Portugal, and between pairs of individuals collected in different countries (e.g. California-Portugal, Scotland-France, etc.). (c) Kinship estimates between heterokaryotic individuals and g21 or g22. Sky-blue dashed lines: kinship = 0.5. Grey dashed lines: kinship = 0.4375 (the threshold used to distinguish immediate kin (parents or offspring) from other kinds of relationships).

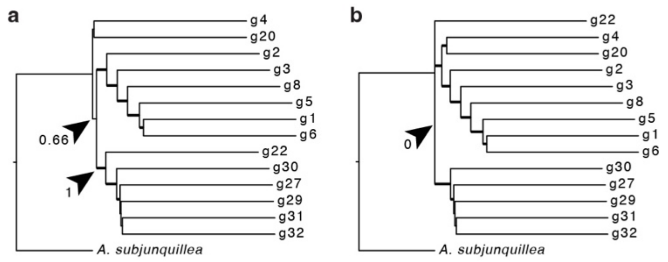

Supplementary Fig. 2. Coalescent-based trees of *A. phalloides* individuals. Phylogenies were reconstructed with ASTRAL unconstrained (a) or constrained by forcing g22 to be the outgroup of all other *A. phalloides* (b). Arrow heads mark branches leading to g22 (a) or the branch between g22 and other *A. phalloides* (b). Numbers indicate local posterior probabilities. There was no support for individual g22 as a sister group of all other *A. phalloides* individuals, hence no support for the hypothesis of g22 (or g21) as a separate species. Branches with local posterior probabilities higher than 0.8 are thickened. Note the terminal branch lengths are forced to one in ASTRAL.

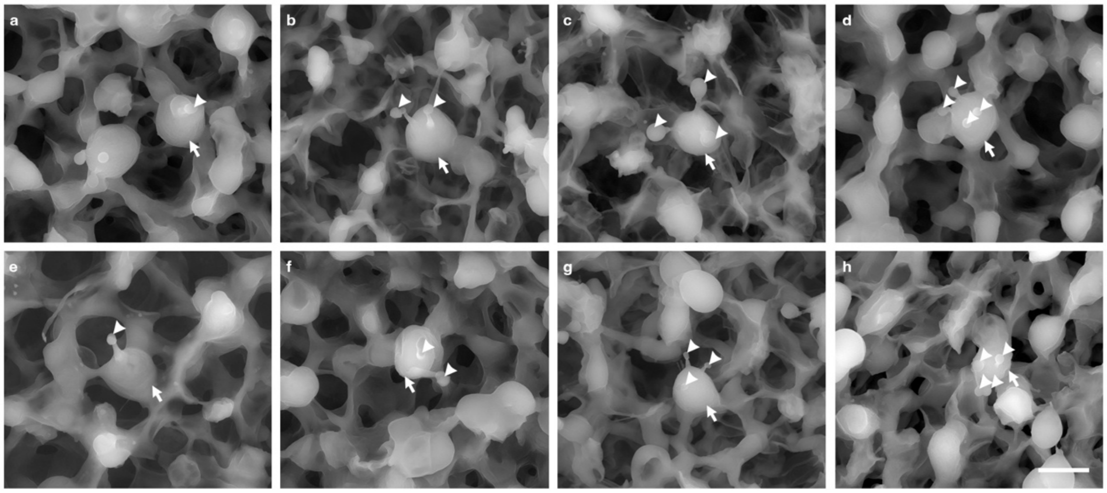

Supplementary Fig. 3. Scanning electron microscopy of basidia. (a–d) 1- to 4-spored basidia in heterokaryotic sporocarps. (e–h) 1- to 4-spored basidia in homokaryotic sporocarps. Arrowheads: immature spores; arrows: basidia. Scale bar: 10 µm.

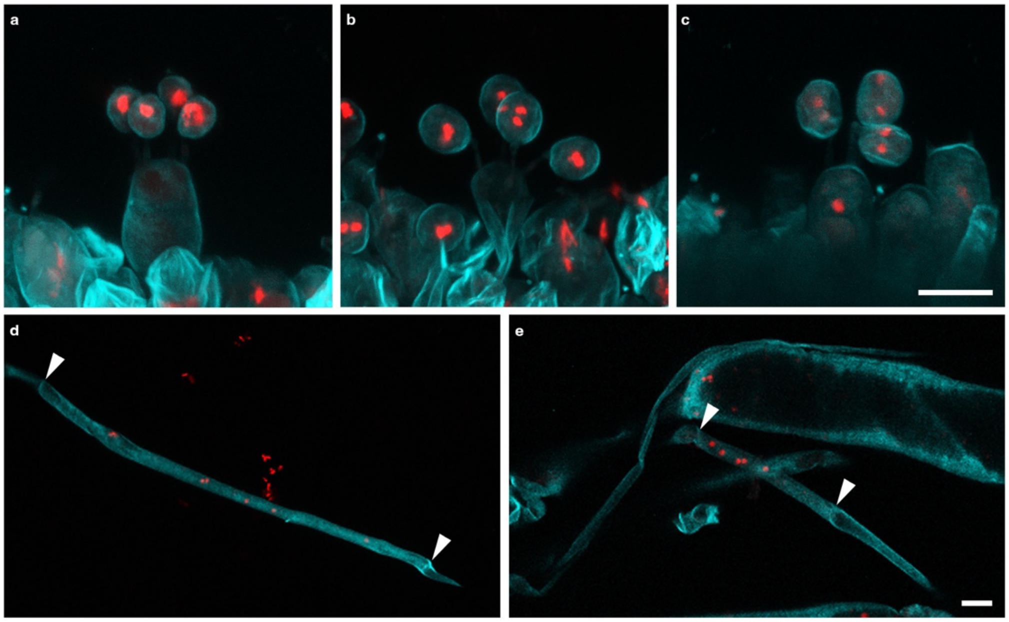

Supplementary Fig. 4. Confocal microscopy of basidia and hyphae. Composite images were created with Z-stack. (a–b) 4-spored basidia of heterokaryotic sporocarps. (c) 3-spored basidia of a heterokaryotic sporocarp. (d–e) Hyphae in the piths of stipes of heterokaryotic (d) and homokaryotic (e) sporocarps. Note the lack of clamp connections. Red: Vybrant Orange (nuclei); cyan: Calcofluor White (cell wall). Scale bar: 10 µm. Arrowheads: septae.

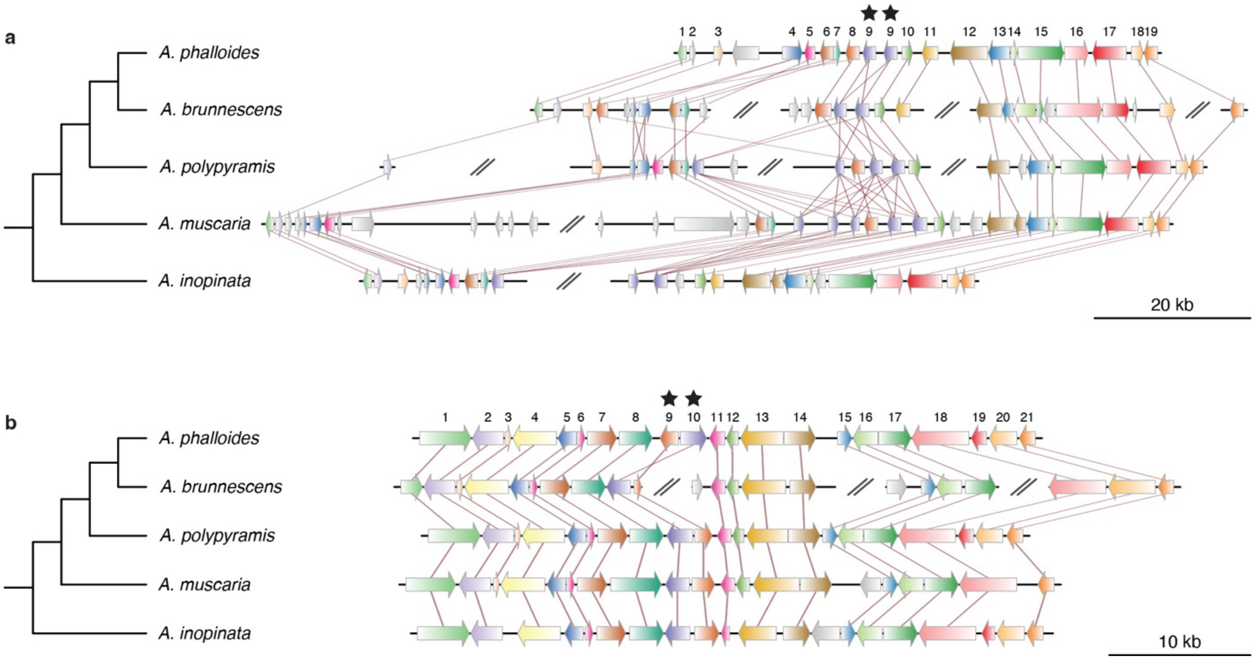

Supplementary Fig. 5. Synteny of P/PR (a) and HD (b) loci across *Amanita* species. The relatively strong synteny in the HD locus was consistent with findings from other species. Putative mating type determining genes (*PR*s and *HD*s) are labeled with stars. On the P/PR locus, twenty genes are homologous to genes in other *Amanita* species, and are functionally annotated as: (1) Cyanamide hydratase, (2) Hypothetical, (3) Hypothetical, (4) NUDIX hydrolase, (5) Nucleotide exchange factor Fes1, (6) Hypothetical, (7) Hypothetical, (8) Leucine-rich repeat domain superfamily, (9) GPCR fungal pheromone mating factor, (10) Hypothetical, (11) Cytochrome P450, (12) Proteasome non-ATPase regulatory subunit 13, (13) Hypothetical, (14) Prefoldin subunit 3, (15) Helicase superfamily 1/2, ATP-binding domain, (16) Meiotically up-regulated protein Msb1/Mug8, (17) UDP-Glycosyltransferase/glycogen phosphorylase, (18) Hypothetical, and (19) Eukaryotic translation initiation factor 2 subunit 3. On HD locus, twenty-one genes are homologous to genes in other *Amanita* species, and are functionally annotated as: (1) Glycine dehydrogenase, (2) Pentatricopeptidase repeat-containing protein, (3) Sec61p translocation complex subunit, (4) Hypothetical, (5) DUF1751, (6) DUF1754, (7) Hypothetical, (8) Mitochondrial intermediate peptidase, (9) HD2, (10) HD1, (11) Beta-flanking protein, (12)

Hypothetical, (13) Glycosyltransferase family 8, (14) ABC1-domain-containing protein, (15) Mitotic spindle assembly checkpoint protein, (16) Nexin sorting protein, (17) Lung seven transmembrane receptor-domain-containing protein, (18) RPB2, (19) Fructosamine kinase, (20) MFS general substrate transporter, and (21) NADP dehydrogenase 1 alpha subcomplex subunit 9.

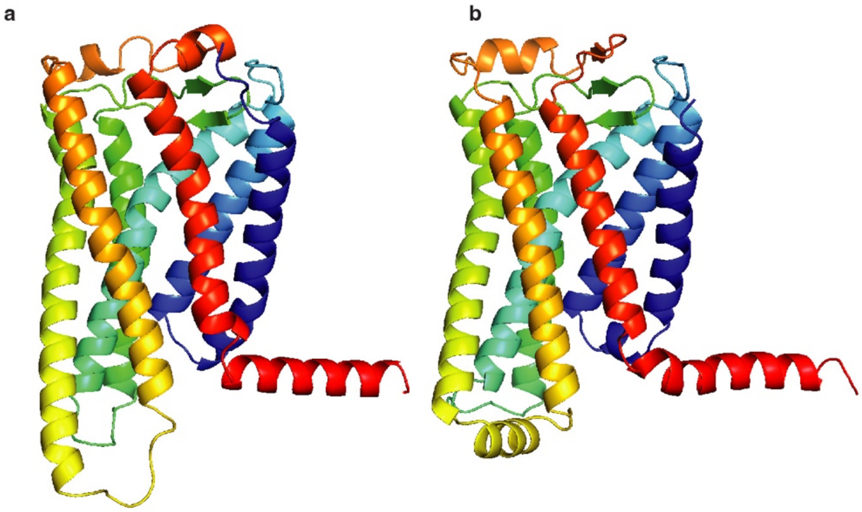

Supplementary Fig. 6. Protein structures predicted for two putative *PR* genes. Each shows similarity to the canonical structure of a GPCR (the protein family of *PR* genes): (a) *Ap.00g075660*, (b) *Ap.*00g075670. Low confidence regions are not shown.

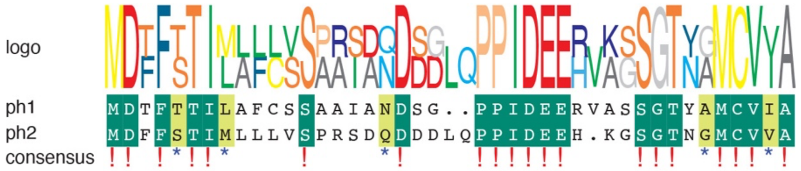

Supplementary Fig. 7. Putative pheromone precursors. The two pheromones both show the presence of a -CxxA motifs, characteristic of fungal pheromones. Neither gene was found near a *PR* gene.

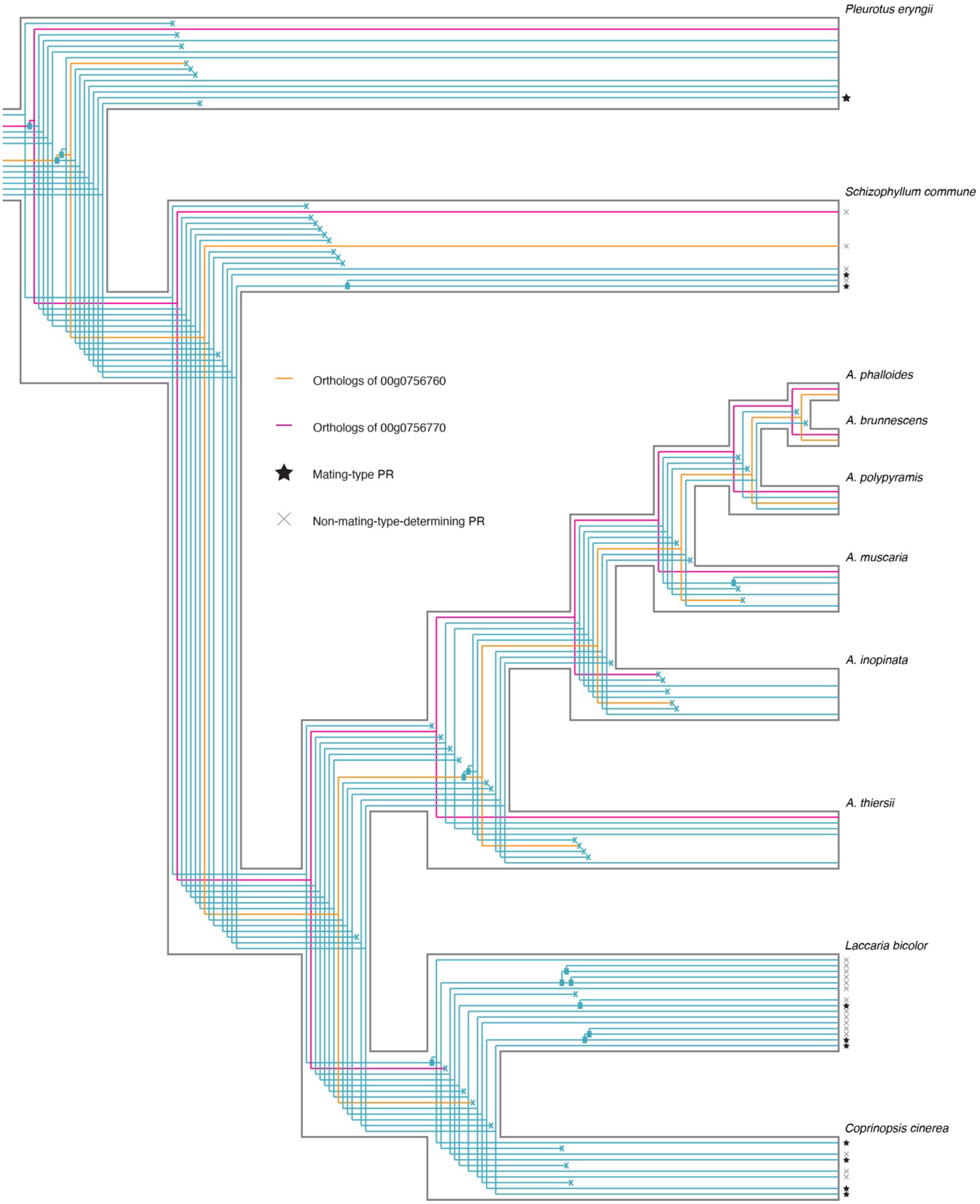

Supplementary Fig. 8. Species-tree-aware protein phylogeny of PRs. The phylogeny demonstrates orthology between PRs of *A. phalloides* and non-mating type determining PRs from species of Agaricales. Note: older duplications may not indicate a duplication within a

genome, instead, the duplication may represent a divergence between alleles in different mating types.

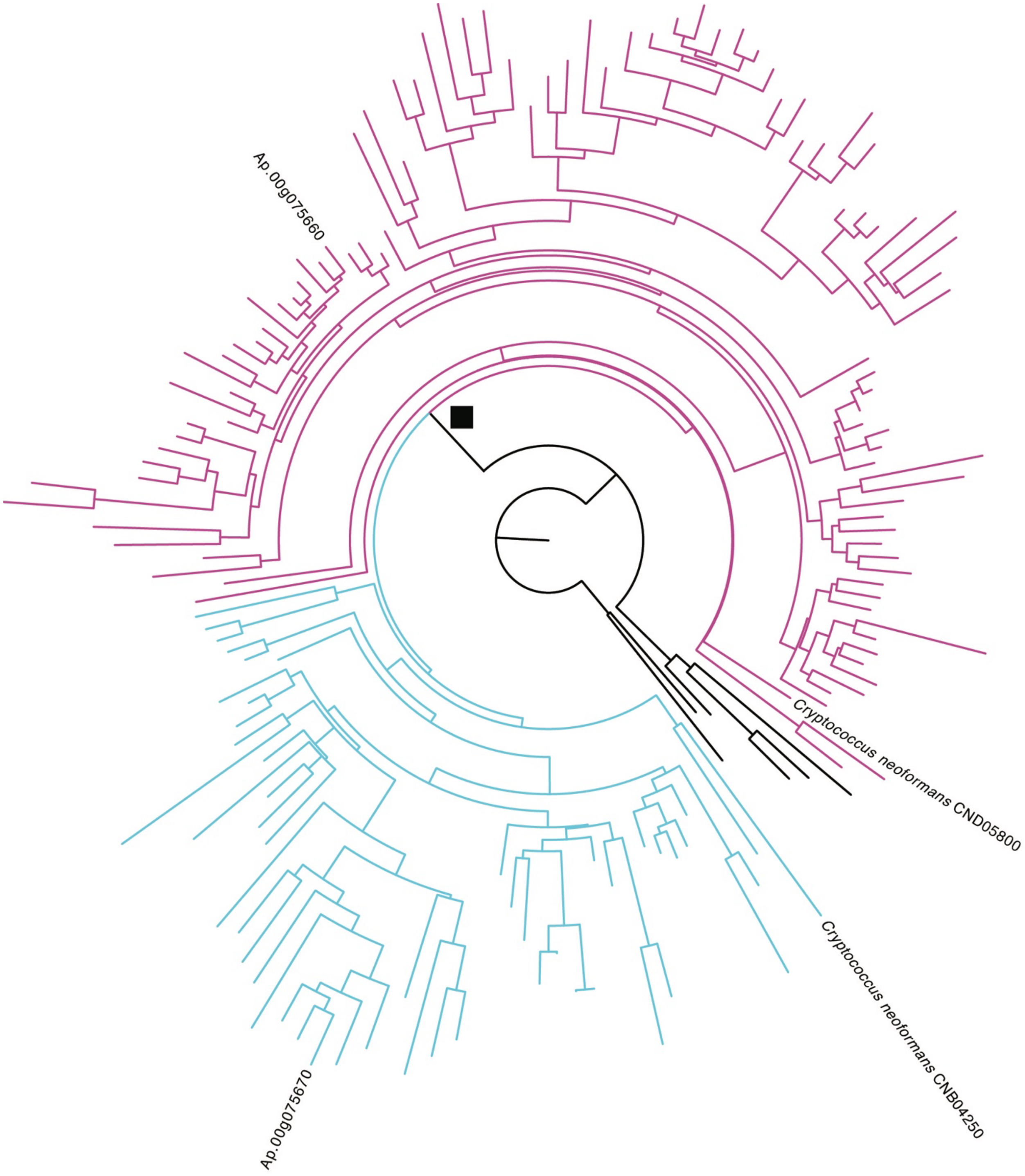

Supplementary Fig. 9. Full species-tree-aware protein phylogeny of PRs. The phylogeny demonstrates orthology between PRs of *A. phalloides* and of *Cryptococcus neoformans*. CND05800: mating type determining (Cpr2); CNB04250: non-mating type determining. Square: most recent common ancestor of the two PRs in *A. phalloides*.

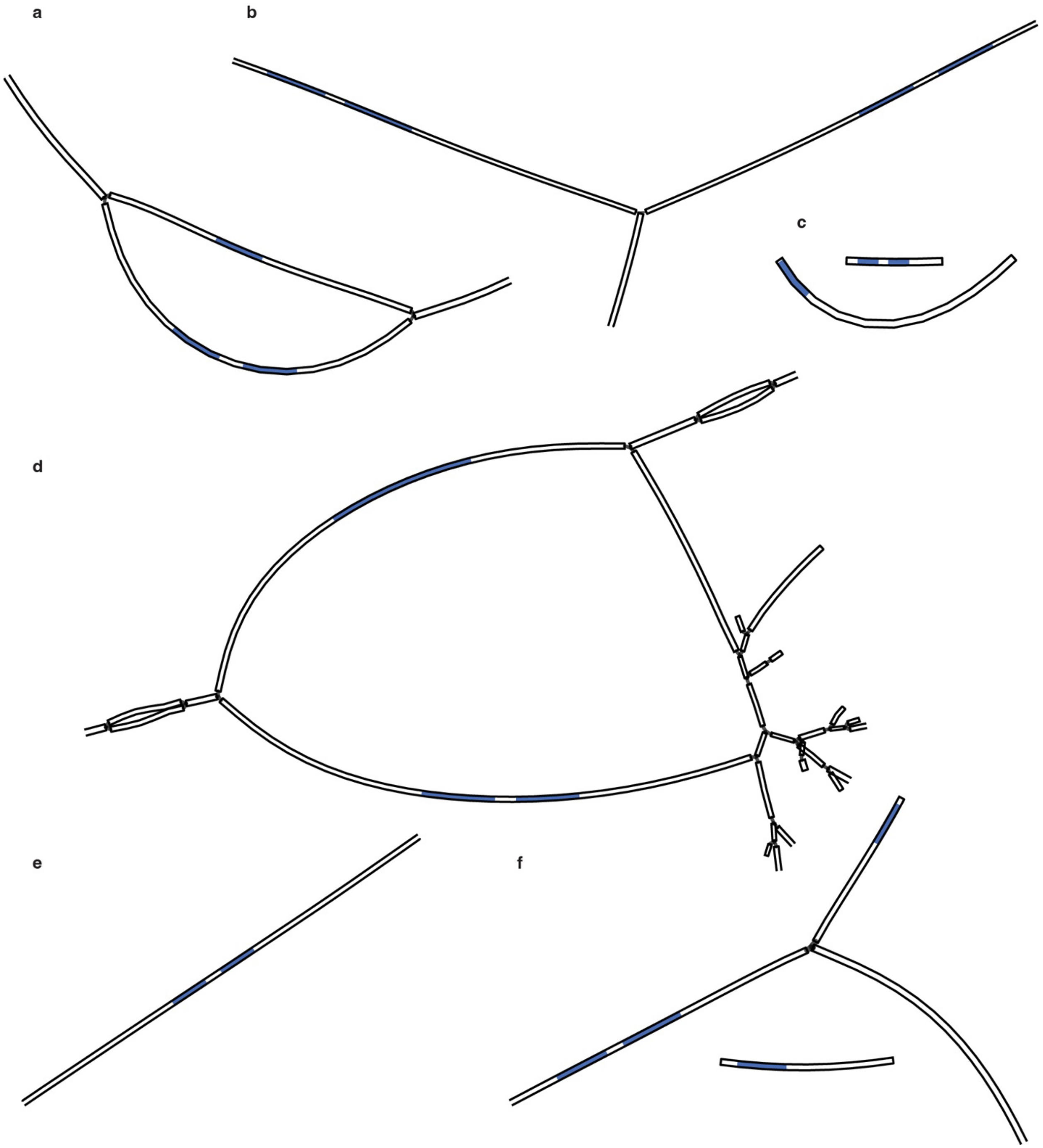

Supplementary Fig. 10. Different assembly string graphs of HD locus. (a) closed bubble (sample 10711), (b) open bubble (sample 10720), (c) detached (sample 10309), (d) complexed (sample 10511), (e) one unitig (sample 10303), and (f) three unitigs (sample 10240). Blue: BLAST hits with HD locus of reference genome as query.

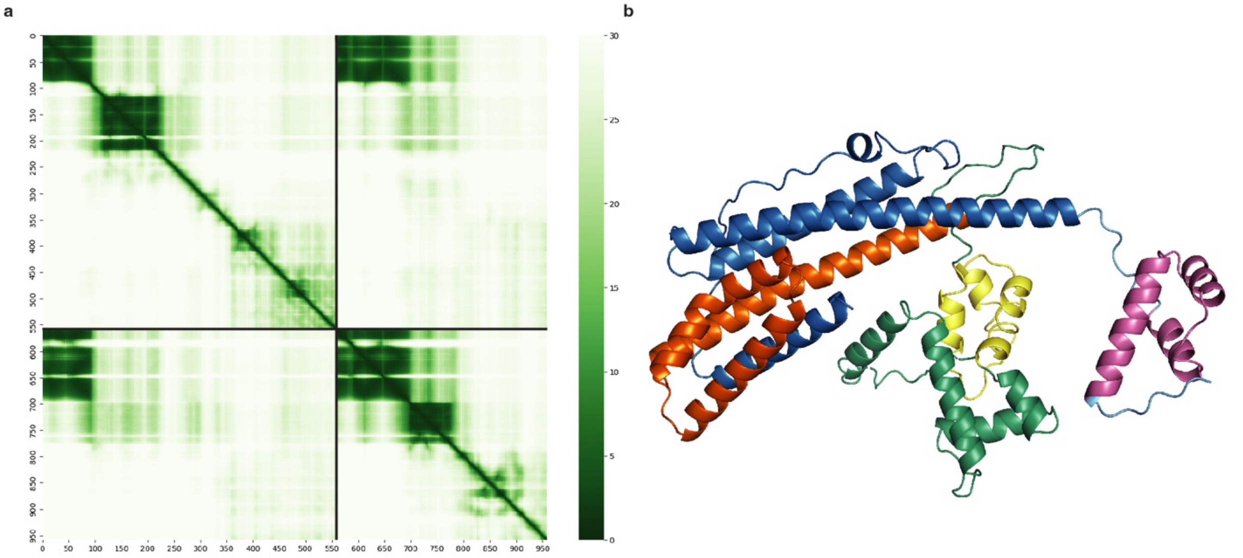

Supplementary Fig. 11. Predicted heterodimer structures of HD1-8 and HD2-5. N-terminals as binding sites and the two homeodomain motifs are highly supported. (a) Predicted alignment errors (PAE) between each pair of amino acids. HD1-8: 1–559; HD2-5: 560–959. Note the low PAE between N-terminals of the two proteins. (b) Cartoon of the heterodimer structure of HD1-8 (orange, green and yellow) and HD2-5 (blue, cyan and magenta). Low confidence regions are not shown. Orange and blue: putative binding domain; yellow and magenta: homeodomains; green and cyan: low confidence regions.

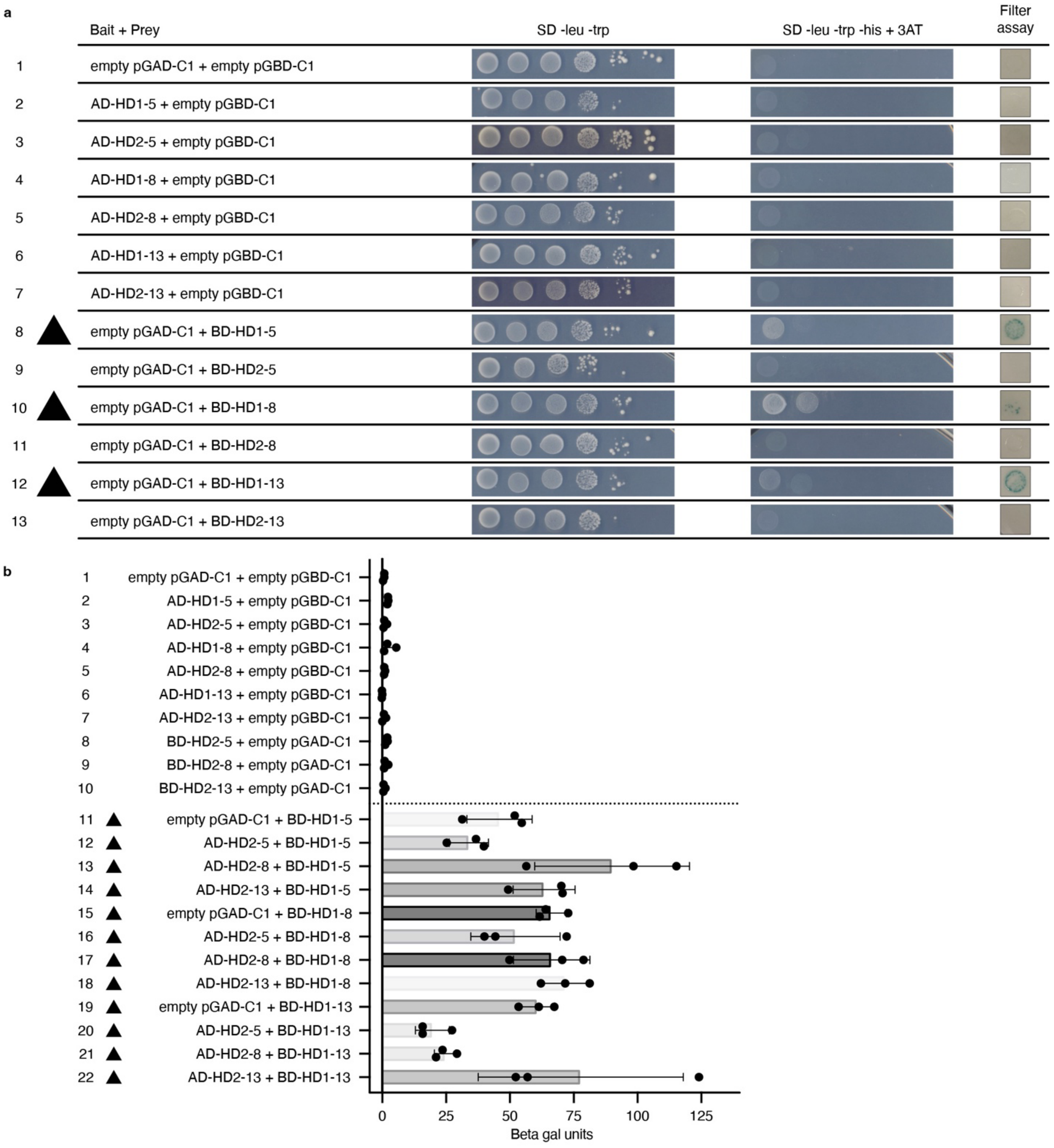

Supplementary Fig. 12. Yeast growth and reporter activity of control strains. (a) Cells at the same starting concentration were 10-fold serially diluted and plated on selective plates. One representative transformant is shown for each interaction tested. Yeast growth was assessed on SD -leu -trp plates. Activity of the reporter gene *pGAL1-HIS3* was assessed by growth on SD -leu -trp -his + 3AT plates. Activity of the reporter gene *pGAL7-LacZ* was determined by filter

assays and (b) liquid β-galactosidase assays. For each interaction, values for three independent biological replicates are shown (black dots). Each dot represents an average value of three technical replicates. Triangles represent reporter activity due to BD-HD1 auto-activation. Error bars represent standard error of the mean (n=3).

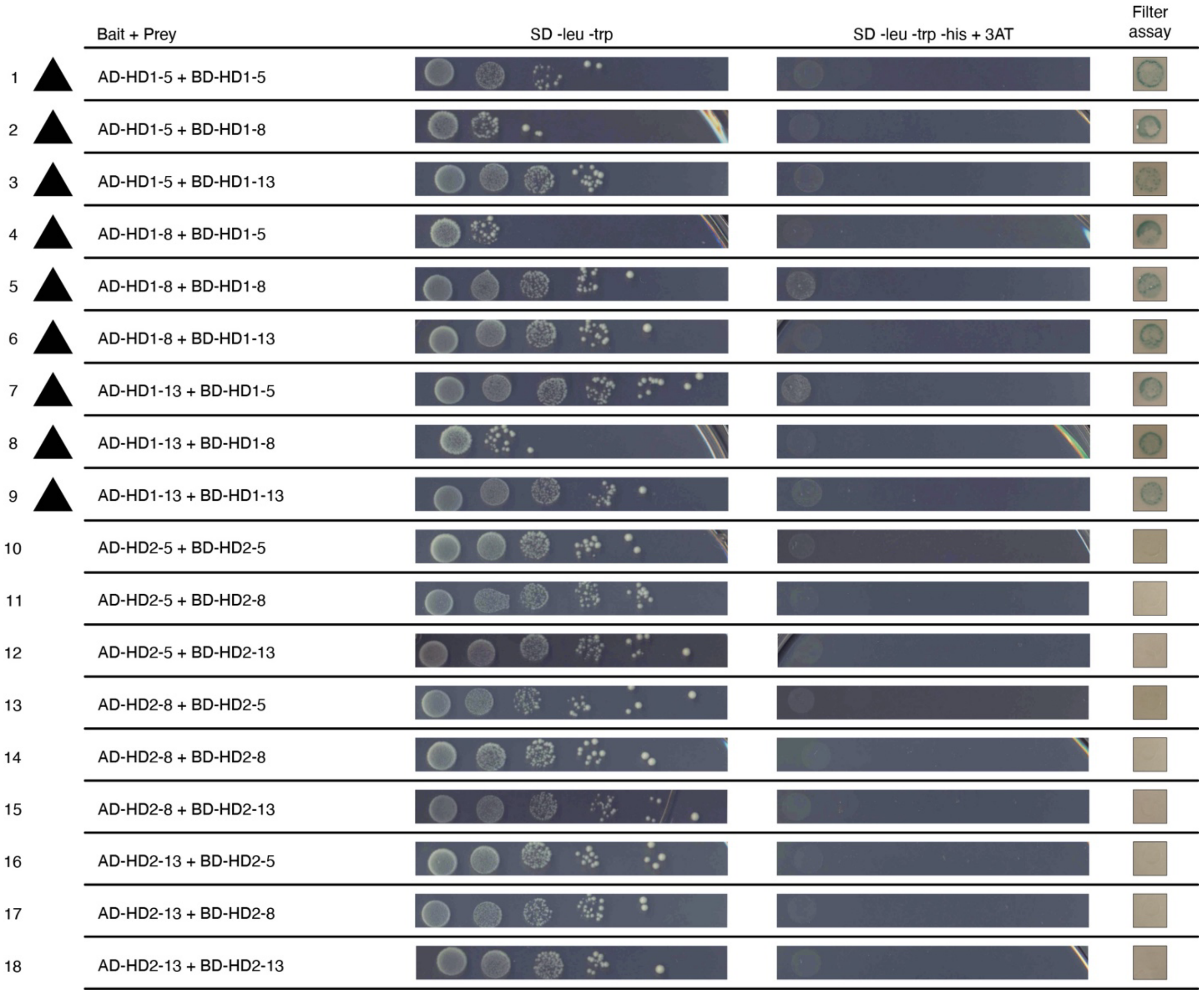

Supplementary Fig. 13. Yeast growth and reporter activity of potential homodimer interactions. Cells at the same starting concentration were 10-fold serially diluted and plated on selective plates. One representative transformant is shown for each potential interaction. Yeast growth was assessed on SD -leu -trp plates. Activity of the reporter gene *pGAL1-HIS3* was assessed by growth on SD -leu -trp -his + 3AT plates. Activity of the reporter gene *pGAL7-LacZ* was determined by filter assays. Triangles represent reporter activity due to BD-HD1 auto-activation.

Supplementary Tables

Supplementary Table 1. Specimens for rediscovering g21 and g22 homokaryotic individuals

ID	Year	Latitude	Longitude	Country	Region	County	City	Site Name
20009	2021	38.0552	-122.8333	USA	CA	Marin	Point Reyes National Seashore	Drake2
20010	2021	38.0550	-122.8333	USA	CA	Marin	Point Reyes National Seashore	Drake2
20011	2021	38.0550	-122.8333	USA	CA	Marin	Point Reyes National Seashore	Drake2
20012	2021	38.0550	-122.8334	USA	CA	Marin	Point Reyes National Seashore	Drake2
20013	2021	38.0546	-122.8331	USA	CA	Marin	Point Reyes National Seashore	Drake2
20014	2021	38.0547	-122.8331	USA	CA	Marin	Point Reyes National Seashore	Drake2
20015	2021	38.0546	-122.8333	USA	CA	Marin	Point Reyes National Seashore	Drake2
20016	2021	38.0548	-122.8331	USA	CA	Marin	Point Reyes National Seashore	Drake2
20017	2021	38.0547	-122.8332	USA	CA	Marin	Point Reyes National Seashore	Drake2
20018	2021	38.0545	-122.8331	USA	CA	Marin	Point Reyes National Seashore	Drake2
20030	2021	38.0547	-122.8330	USA	CA	Marin	Point Reyes National Seashore	Drake2
20032	2021	38.0550	-122.8374	USA	CA	Marin	Point Reyes National Seashore	Drake4
20033	2021	38.0550	-122.8371	USA	CA	Marin	Point Reyes National Seashore	Drake4
20034	2021	38.0552	-122.8355	USA	CA	Marin	Point Reyes National Seashore	Drake4
20036	2021	38.0547	-122.8374	USA	CA	Marin	Point Reyes National Seashore	Drake4
20041	2021	38.0546	-122.8333	USA	CA	Marin	Point Reyes National Seashore	Drake0
20043	2021	38.0544	-122.8334	USA	CA	Marin	Point Reyes National Seashore	Drake0
20046	2021	38.0553	-122.8329	USA	CA	Marin	Point Reyes National Seashore	Drake2
20047	2021	38.0552	-122.9329	USA	CA	Marin	Point Reyes National Seashore	Drake2
20048	2021	38.0553	-122.8329	USA	CA	Marin	Point Reyes National Seashore	Drake2
20049	2021	38.0554	-122.8329	USA	CA	Marin	Point Reyes National Seashore	Drake2
20050	2021	38.0549	-122.8330	USA	CA	Marin	Point Reyes National Seashore	Drake2
20051	2021	38.0548	-122.8330	USA	CA	Marin	Point Reyes National Seashore	Drake2
20052	2021	38.0546	-122.8327	USA	CA	Marin	Point Reyes National Seashore	Drake2
20053	2021	38.0546	-122.8328	USA	CA	Marin	Point Reyes National Seashore	Drake2
20054	2021	38.0545	-122.8330	USA	CA	Marin	Point Reyes National Seashore	Drake2
20055	2021	38.0546	-122.8326	USA	CA	Marin	Point Reyes National Seashore	Drake2
20056	2021	38.0546	-122.8326	USA	CA	Marin	Point Reyes National Seashore	Drake2
20058	2021	38.0547	-122.8325	USA	CA	Marin	Point Reyes National Seashore	Drake2
20060	2021	38.0547	-122.8323	USA	CA	Marin	Point Reyes National Seashore	Drake2

Supplementary Table 2. Primers used for detecting heterozygosity in specimens without whole genome sequencing data

Primer ID	Forward	Reverse	Amplicon Length	Contig	Start	End	SNP sites	Functional Annotation	Nick Name	P(hom) (CA)*	P(hom) (All)*
00g060260-3	ACC GCC AGA CTT GTC AAA CA	TCA ATC GCA GCA GTG GAA CT	691	Contig87	400939	401629	multiple	beta-flanking gene		0.13†	0.15†
00g060260-16	CAG CCT GGA CAA TCT CGT CA	TCA ATC GCA GCA GTG GAA CT	248	Contig87	401382	401629	multiple	beta-flanking gene		0.31†	0.37†
00g000510-2	TTC AGC CGA CGT TAC GAC TC	TGG GAG TTG GCG TTG TTT CT	220	Contig0	327946	328165	328045A/G	Eukaryotic translation initiation factor 3 subunit C	Het-1	0.75	0.57
00g004840-9	AAG CCT AAA GGT GGC GTT CA	GCG AGA TCG ACC GCA TAG TT	137	Contig2	766786	766922	766833G/A; 766848C/T; 766902T/C	Fimbrin	Het-2	0.58	0.43
00g009240-3	GCT ATT GTC CAG GAG GTC CA	GCC TAG ACT TTT CAG CTG CTC	149	Contig5	299743	299891	299822A/C; 299864G/A	Elongator complex protein 3	Het-3	0.55	0.51
00g011220-3	GGA TAT GCG GAC CAG GAT CG	TCG TCC TCG AAG TAA GCA TCC	153	Contig5	748470	748622	748593G/C	Transcription elongation factor Spt6	Het-4	0.31	0.29
00g016590-6	CAC TTT GCG CAG ACT CAC G	GTA AAC AGT CCG AAG GGG CT	172	Contig7	692570	692741	692691T/C	RNA polymerase I-specific transcription initiation factor RRN3	Het-5	0.53	0.52
00g021750-9	AGA TCC CGA AGT TCA CTT CAA A	AGT TCT TGA CCT TCT CTG GGT	116	Contig8	62025	62140	62082T/C	Clathrin heavy chain	Het-6	0.53	0.54
00g023990-5	GCA TCT TCG TAC GGG ACA GT	GCA GCT TGG CTT TCG TCA AT	156	Contig9	207878	208033	207956A/G; 208004A/G	DNA-directed RNA polymerase	Het-7	0.51	0.49
00g053490-1	ATC TCA CAC AGC CAG GTT GG	TAG GGA TGA CCC TCC TTC GG	133	Contig63	110061	110193	110102G/A; 110119T/C	DNA polymerase gamma	Het-9	0.52	0.50
00g072790-8	TCC AAG AAC AAT GAC CGG GAA	ATC TCG GAG TCG GTT CCT TTG	143	Contig104	676464	676606	676503G/A; 676536G/A	GPI-anchored wall transfer protein	Het-10	0.47	0.43
00g083180-4	AAC GAA GTG GAA TGC GCG A	TAG GCC GCA TCA CAC AAG AC	111	Contig166	99533	99643	99599T/G	DNA polymerase alpha subunit B	Het-11	0.51	0.51

* Estimates of probability of homozygosity are from clone corrected dataset, without haploids, of either Californian samples or all samples

† Due to the complexity of these two regions, the probability of homozygosity is estimated with clipping off 80 nucleotides of either ends of the amplicon, others are estimated by the exact observable sites in Sanger sequences

Supplementary Table 3. Plasmids used for yeast two-hybrid assays

Plasmid	Description	Reference
pCH478 (pGAD-C1)	pGAD-C1	James et al., 1996 [5]
pCH312 (pGBD-C1)	pGBD-C1	James et al., 1996 [5]
pGAD-C1-HD1-5	HD1 ORF of allele 5 fused to the *GAL4* activation domain	This study
pGAD-C1-HD1-8	HD1 ORF of allele 8 fused to the *GAL4* activation domain	This study
pGAD-C1-HD1-13	HD1 ORF of allele 13 fused to the *GAL4* activation domain	This study
pGAD-C1-HD2-5	HD2 ORF of allele 5 fused to the *GAL4* activation domain	This study
pGAD-C1-HD2-8	HD2 ORF of allele 8 fused to the *GAL4* activation domain	This study
pGAD-C1-HD2-13	HD2 ORF of allele 13 fused to the *GAL4* activation domain	This study
pGBD-C1-HD1-5	HD1 ORF of allele 5 fused to the *GAL4* DNA binding domain	This study
pGBD-C1-HD1-8	HD1 ORF of allele 8 fused to the *GAL4* DNA binding domain	This study
pGBD-C1-HD1-13	HD1 ORF of allele 13 fused to the *GAL4* DNA binding domain	This study
pGBD-C1-HD2-5	HD2 ORF of allele 5 fused to the *GAL4* DNA binding domain	This study
pGBD-C1-HD2-8	HD2 ORF of allele 8 fused to the *GAL4* DNA binding domain	This study
pGBD-C1-HD2-13	HD2 ORF of allele 13 fused to the *GAL4* DNA binding domain	This study

Supplementary Table 4. Yeast strain used for yeast two-hybrid assays

Strain	Genotype	Reference
CHY1268 (PJ69-4a)	MATa trp1-901 leu2-3, 112 ura3-52 his3-200 gal4*D gal80D LYS::GAL1-HIS3 GAL2-ADE2 met2::GAL7-lacZ*	James et al., 1996 [5]

Supplementary Table 5. SNP genotypes of known and putative homokaryotic specimens

SNP sites	Known		Putative*								
	10224	10293	10028	10038	10044	10048	10051	11108	20018	20030	20034
Contig0:328045A/G	G	G	A	A	A	A	A	A	G	G	G
Contig2:766833G/A	A	G	-	-	-	-	-	-	-	-	-
Contig2:766848C/T	T	C	T	C	C/T	C/T	C	C	C	T	T
Contig2:766902T/C	T	T	T	T	T	T	T	T	T	T	T
Contig5:299822A/C	A	A	A	A	A	A	A	A	A	A	A
Contig5:299864G/A	A	A	A/G	A	A/G	A/G	A/G	A/G	A	A	A
Contig5:748593G/C	C	C	G/C	G/C	G/C	G	G	G/C	C	C	C
Contig7:692691T/C	T	C	C	C	C	C	C	C	C	T	T
Contig8:62082T/C	T	T	C	C	C	C/T	C	C	T	T	T
Contig9:207956A/G	G	A	A	A	A/G	A/G	A	A/G	A	G	G
Contig9:208004A/G	G	A	A	A	A/G	A/G	A	A/G	A	G	G
Contig63:110102G/A	G	A	-	-	-	-	-	-	-	-	-
Contig63:110119T/C	T	C	C	C	C	C/T	T	C	C	T	T
Contig104:676503G/A	G	G	-	-	-	-	-	-	-	G	-
Contig104:676536G/A	G	G	A	A	A	A/G	A/G	A	G	G	G
Contig166:99599T/G	G	T	T	T/G	T	T	T	T/G	T	G	G

* Each specimen possesses a homozygous beta-flanking gene

Supplementary Discussion

Explanations for the absence of heterozygosity

The simplest explanation for an absence of heterozygosity is homokaryosis: individuals house the same haploid genome in every nucleus of the mycelium (either as a single copy (n) or as two copies (2n)). If we are wrong, and some nuclei house one copy of the genome while others house two (i.e. a mix of haploid and diploid nuclei), then the haploid genome would be considered as the unit driving observed phenomena, and statements like "nuclei appeared to have persisted in invaded habitats for at least 17 years" would translate to "haploid genomes appeared to have persisted in invaded habitats for at least 17 years".

Other strategies to identify mating systems

Traditionally, mating systems of fungi are explored with mating experiments, by co-culturing sexual spores from sporocarps and observing subsequent behaviors. However, we cannot adopt this strategy because *A. phalloides* cannot be cultured.

The natural history of the homokaryon g22 does not support an hypothesis of pseudosexuality

Fungal sexual systems are diverse and intensively studied. We did consider a range of alternative hypotheses to explain our data, but none appear to describe the dynamics we observe both in nature and the laboratory. In particular, we considered the hypothesis of pseudosexuality[6]. Pseudosexuality is a process through which only a single nucleus is inherited after bisexual mating. We note pseudosexuality is rare (to date described from a single species), and in the species where it is described it is also rare (involving only 1% of basidia). To explore

whether pseudosexuality can explain our data, we thought through two possibilities involving the distribution of the homokaryotic g22: one pseudosexual event or two pseudosexual events. If one pseudosexual event generated g22, the range of g22 should be similar in size to the sizes of heterokaryotic individuals, because g22 appears to grow like heterokaryotic individuals (the two kinds of mushrooms appear similar, and there is no evidence for asexual fragmentation or sporulation[7]). However, the range of g22 is at least 200 m in diameter. Its size is inconsistent with our data on the sizes of heterokaryotic *A. phalloides* individuals in both North American and Europe. Heterokaryotic individuals are much smaller, suggesting a single pseudosexual event (and subsequent mycelial growth) cannot explain the origin of g22.

Next, we considered an hypothesis of two pseudosexual events. Because pseudosexuality appears very rare, it would be very unlikely for the same nucleus to emerge twice at two different sites (and be collected by us both times). Therefore, pseudosexuality does not emerge as a likely explanation for the discovery of homokaryotic *A. phalloides* sporocarps.

Exploration of pheromone transporters

During peer review, we were asked to search for the presence of orthologs of the STE6 pheromone transporter, which is required to secrete pheromones in *Cr. neoformans*[8]. We attempted to identify an ortholog of STE6 in *A. phalloides* with BLAST. We identified 27 genes as potentially homologous. One gene (*Ap.00g014230*) has a significantly higher identity to the *Cr. neoformans* STE6 (45.83%) than the others and was functionally annotated as a type-1 ABC transporter, but it is hard to interpret the function of this gene in *A. phalloides* without genetic tools, in part because pheromone secretion in at least one other Agaricales is independent of STE6[9].

Supplementary References

1. Rutz, C., Klein, W. & Schülein, R. Chapter twelve - N-terminal signal peptides of G protein-coupled receptors: significance for receptor biosynthesis, trafficking, and signal transduction. in *Progress in Molecular Biology and Translational Science* (ed. Wu, G.) vol. 132 267–287 (Academic Press, 2015).
2. Brown, A. J. & Casselton, L. A. Mating in mushrooms: increasing the chances but prolonging the affair. *Trends Genet.* **17**, 393–400 (2001).
3. Kohler, A. *et al.* Convergent losses of decay mechanisms and rapid turnover of symbiosis genes in mycorrhizal mutualists. *Nat. Genet.* **47**, 410–415 (2015).
4. Chen, B. *et al.* Fruiting body formation in *Volvariella volvacea* can occur independently of its MAT-A-controlled bipolar mating system, enabling homothallic and heterothallic life cycles. *G3-Genes Genom. Genet.* (2016) doi:10.1534/g3.116.030700.
5. James, P., Halladay, J. & Craig, E. A. Genomic libraries and a host strain designed for highly efficient two-hybrid selection in yeast. *Genetics* **144**, 1425–1436 (1996).
6. Yadav, V., Sun, S. & Heitman, J. Uniparental nuclear inheritance following bisexual mating in fungi. *eLife* **10**, e66234 (2021).
7. Hutchison, L. J. Absence of conidia as a morphological character in ectomycorrhizal fungi. *Mycologia* **81**, 587–594 (1989).
8. Hsueh, Y.-P. & Shen, W.-C. A homolog of Ste6, the a-Factor transporter in *Saccharomyces cerevisiae*, is required for mating but not for monokaryotic fruiting in *Cryptococcus neoformans*. *Eukaryot. Cell* **4**, 147–155 (2005).

9. Fowler, T. J., DeSimone, S. M., Mitton, M. F., Kurjan, J. & Raper, C. A. Multiple sex pheromones and receptors of a mushroom-producing fungus elicit mating in yeast. *Mol. Biol. Cell* **10**, 2559–2572 (1999).
