# Supplementary material for: Invasive Californian death caps develop mushrooms unisexually and bisexually
Source: Nat Commun. 2023 Oct 24;14:6560. doi: 10.1038/s41467-023-42317-z (PMC10598064; doi:10.1038/s41467-023-42317-z)
Supplement: Supplementary file 3 — Description of Additional Supplementary Files [file 41467_2023_42317_MOESM3_ESM.pdf]

Description of Additional Supplementary Files

Supplementary Data 1
Description: Specimens with whole genome sequencing data

Supplementary Data 2
Description: Additional specimens without whole genome sequencing data screened as potentially homokaryotic sporocarps
