# Supplementary material for: Invasive Californian death caps develop mushrooms unisexually and bisexually
Source: Nat Commun. 2023 Oct 24;14:6560. doi: 10.1038/s41467-023-42317-z (PMC10598064; doi:10.1038/s41467-023-42317-z)
Supplement: Supplementary file 5 — Reporting Summary [file 41467_2023_42317_MOESM5_ESM.pdf]

nature portfolio

Corresponding author(s): Yen-Wen Wang

Last updated by author(s): 08/24/2023

Reporting Summary

Nature Portfolio wishes to improve the reproducibility of the work that we publish. This form provides structure for consistency and transparency in reporting. For further information on Nature Portfolio policies, see our Editorial Policies and the Editorial Policy Checklist.

Statistics

For all statistical analyses, confirm that the following items are present in the figure legend, table legend, main text, or Methods section.

n/a	Confirmed	
☐	☒	The exact sample size (n) for each experimental group/condition, given as a discrete number and unit of measurement
☐	☒	A statement on whether measurements were taken from distinct samples or whether the same sample was measured repeatedly
☐	☒	The statistical test(s) used AND whether they are one- or two-sided<br>*Only common tests should be described solely by name; describe more complex techniques in the Methods section.*
☒	☐	A description of all covariates tested
☒	☐	A description of any assumptions or corrections, such as tests of normality and adjustment for multiple comparisons
☐	☒	A full description of the statistical parameters including central tendency (e.g. means) or other basic estimates (e.g. regression coefficient) AND variation (e.g. standard deviation) or associated estimates of uncertainty (e.g. confidence intervals)
☐	☒	For null hypothesis testing, the test statistic (e.g. F, t, r) with confidence intervals, effect sizes, degrees of freedom and P value noted<br>*Give P values as exact values whenever suitable.*
☒	☐	For Bayesian analysis, information on the choice of priors and Markov chain Monte Carlo settings
☒	☐	For hierarchical and complex designs, identification of the appropriate level for tests and full reporting of outcomes
☒	☐	Estimates of effect sizes (e.g. Cohen's d, Pearson's r), indicating how they were calculated

Our web collection on statistics for biologists contains articles on many of the points above.

Software and code

Policy information about availability of computer code

Data collection	No software was used to collect data.
Data analysis	All programs used to analyze data are described in Methods and citations to relevant publications (and programs) provided. Custom codes are deposited in an Open Science Framework created by us (10.17605/OSF.IO/BQ2RU and 10.17605/OSF.IO/KDE9C). We have also provided the necessary information to access our OSF within the manuscript itself.

For manuscripts utilizing custom algorithms or software that are central to the research but not yet described in published literature, software must be made available to editors and reviewers. We strongly encourage code deposition in a community repository (e.g. GitHub). See the Nature Portfolio guidelines for submitting code & software for further information.

Data

Policy information about availability of data

All manuscripts must include a data availability statement. This statement should provide the following information, where applicable:

- Accession codes, unique identifiers, or web links for publicly available datasets
- A description of any restrictions on data availability
- For clinical datasets or third party data, please ensure that the statement adheres to our policy

The raw genomic reads we used are accessible in a Sequence Read Archive (SRA) BioProject PRJNA565149. Raw transcriptomic reads are accessible through the SRA BioProject PRJNA689850.

Research involving human participants, their data, or biological material

Policy information about studies with human participants or human data. See also policy information about sex, gender (identity/presentation), and sexual orientation and race, ethnicity and racism.

Reporting on sex and gender	NA
Reporting on race, ethnicity, or other socially relevant groupings	NA
Population characteristics	NA
Recruitment	NA
Ethics oversight	NA

Note that full information on the approval of the study protocol must also be provided in the manuscript.

Field-specific reporting

Please select the one below that is the best fit for your research. If you are not sure, read the appropriate sections before making your selection.

☐ Life sciences ☐ Behavioural & social sciences ☒ Ecological, evolutionary & environmental sciences

For a reference copy of the document with all sections, see nature.com/documents/nr-reporting-summary-flat.pdf

Ecological, evolutionary & environmental sciences study design

All studies must disclose on these points even when the disclosure is negative.

Study description	We use genomes generated from mushrooms of Amanita phalloides to describe the natural history and in particular the reproductive strategies of the fungus in both native and invasive ranges.
Research sample	The samples are a collection of sporocarps (mushrooms). The majority were collected in California, representing an invasive range, but we also characterized additional mushrooms from Europe, representing native ranges. Mushrooms are ephemeral structures and we sampled whatever we could find, choosing undamaged sporocarps and sampling all available mushrooms when populations were relatively small (e.g. a dozen or fewer mushrooms) and choosing mature, large mushrooms when populations were large (e.g. numbering nearly 100). In each case we made the best decisions we could in the field as we encountered the fungus. If we left mushrooms behind (if we didn't sample every mushroom), in addition to not choosing damaged mushrooms, we often chose mushrooms spaced apart from each other, in other words we typically did not choose two mushrooms if they were adjacent/growing in the same spot. We emphasize our decisions were made in the field and apart from general philosophical discussions ("if we have to choose, let's not choose mushrooms growing as a cluster") we did not travel with a specific plan as to how many mushrooms we would find in each spot.
Sampling strategy	We describe an unexpected and opportunistic finding, unisexual as well as bisexual reproduction in Amanita phalloides, and we did not plan our sampling strategy with unisexuality in mind. However, we did design our sampling with the aim of discovering the mechanisms used by death caps to invade. For this reason we visited and disproportionately sampled from the same sites in California over multiple years (with permits). As context for our Californian collections, we used mushrooms sent to us by European collaborators (who also collect with permits), as well as tissues sampled by herbarium curators from herbarium collections (e.g., the death cap collection at Kew). We did not choose a specific sampling size in advance. Eventually, we generated genomes from as many mushrooms as we could afford to characterize. When choosing which mushrooms to sequence, our criteria included the quality of the DNA as well as the PI (Anne Pringle's) sense of how many mushrooms we should pick from each population. Pringle's decisions were based on her experiences in the field and intuition as to which populations would be most interesting, and her sense of how to build a comparative dataset.
Data collection	Sporocarps were harvested and kept either as dried samples or frozen in buffer prior to DNA extraction. In the case of herbarium specimens sent to us by curators, metadata were drawn from herbarium labels and we do not know how the various collectors collected the information found on labels (but probably it was pen/pencil and paper). Metadata collected by us were collected in the field using pen/pencil and paper and recorded by the individual collecting the mushrooms. In California in 2004 and 2015, Pringle was either the collector or with the collector and in California in 2014, Pringle supervised remotely. Portuguese mushrooms were collected by Susana Goncalves. Data collection from sequenced genomes is described in our manuscript.
Timing and spatial scale	Please see Supplementary Tables 1, 2 and 6.<br>START date for mushroom collecting: 16 November 2004. STOP date for mushroom collecting: 19 December 2021. The frequency and periodicity of mushroom collecting are described in our manuscript, as are the dates and location of each collected mushroom. As described above, we had no master plan for where or when to collect, and collected opportunistically based on where we could find collaborators and the funds (and time) available for travel to field sites.<br>GENOMES: In California, sporocarps were collected in 2004, 2014 and 2015 at two sites in Point Reyes National Seashore, California (the two sites are 100 m away from each other) and three sites in Portugal (~20 km away from each other), but additional herbarium samples were also included in our analyses, and these date back to 1978 for the oldest specimen, and herbarium samples are from

	sites scattered across Europe. ADDITIONAL SAMPLES FOR SANGER SEQUENCING were collected in 2021 at three sites in California; one original site (Drake 2) and two additional sites (Drake 0 and Drake 4; ~200 m away from Drake 2). Additional herbarium samples collected between 1994 and 2019 were also characterized using Sanger sequencing.
Data exclusions	Two sequenced samples generated poor quality genomes and we did not use the genomes in analyses. These exclusion criteria were not pre-established.
Reproducibility	To verify our discovery of unisexuality as real and reproducible, we traveled back to our Californian field sites in December 2021 and searched for additional unisexual mushrooms. Additional unisexual mushrooms were recovered from the same sites we had sampled in 2004, 2014 and 2015, and these mushrooms belonged to both of the unisexual individuals we had already found from 2004, 2014 and 2015. The lack of heterozygosity in the newly collected unisexual sporocarps was confirmed with Sanger sequencing. Given the longevity of the individuals, we anticipate anyone could visit the sites to harvest unisexual mushrooms for themselves, if they wished!
Randomization	Randomization is not relevant to this study because it is an observational study based in the natural habitats of the fungus, reliant on finding the mushrooms where they grow.
Blinding	Blinding is not relevant to this study because there is no subjective judgment involved in categorizing mushrooms as unisexual or bisexual; the data are publicly available and anyone can go and repeat the analyses we have undertaken (using the code we have provided in our OSF, or by writing their own code). Any new analysis would sort the mushrooms into the same categories we have used, either unisexual or bisexual according to the mating system found in each mushroom.

Did the study involve field work? [x] Yes [ ] No

Field work, collection and transport

Field conditions	Field work at Point Reyes National Seashore was conducted in November and December of 2004, 2014, 2015 and 2021.<br>Field work in Portugal was conducted in November of 2015. While the environments of each site are likely directly relevant to the presence of the fungus (it can only grow in certain environments and with certain hosts), we do not think the environmental variables of either site (each site's temperature, rainfall, plants, etc.) are relevant to the discovery of unisexuality, which is a finding based on the genetics of the different populations.
Location	Field work at Point Reyes National Seashore was at or near 38°03'18"N, 122°50'01"W. Field work in Portugal was at or near 40° 07'20"N 8°12'35"W, 40°12'45"N 8°27'01"W and 40°27'27"N, 8°46'07"W. See manuscript for detailed location information for each collected mushroom.
Access & import/export	Field work at Point Reyes National Seashore was conducted under permits granted to the Bruns and Pringle laboratories (see acknowledgements). Field work in Portugal was conducted under permits granted to S. Goncalves. Anne Pringle has permission to import species of Amanita to her lab, granted from the USDA/APHIS. Herbarium collections were governed by individual agreements (typically, email exchanges) with herbaria.
Disturbance	Sporocarps were carefully collected from the ground with minimal disturbance to soil.

Reporting for specific materials, systems and methods

We require information from authors about some types of materials, experimental systems and methods used in many studies. Here, indicate whether each material, system or method listed is relevant to your study. If you are not sure if a list item applies to your research, read the appropriate section before selecting a response.

Materials & experimental systems

n/a	Involved in the study
[x]	[ ] Antibodies
[x]	[ ] Eukaryotic cell lines
[x]	[ ] Palaeontology and archaeology
[x]	[ ] Animals and other organisms
[x]	[ ] Clinical data
[x]	[ ] Dual use research of concern
[x]	[ ] Plants

Methods

n/a	Involved in the study
[x]	[ ] ChIP-seq
[x]	[ ] Flow cytometry
[x]	[ ] MRI-based neuroimaging
